# Hyperuricemia in ob/ob mice relates to hepatocellular pyruvate metabolism/ xanthine oxidase axis

**DOI:** 10.1371/journal.pone.0328794

**Published:** 2025-08-06

**Authors:** Shan Zhang, Shasha Hu, Haibo Tan, Ertao Jia

**Affiliations:** 1 Shenzhen Traditional Chinese Medicine Hospital Affiliated to Nanjing University of Chinese Medicine, Shenzhen, PR China; 2 Shenzhen Traditional Chinese Medicine Hospital, Department of Rheumatism, Shenzhen, PR China; 3 The Fifth Clinical College of Guangzhou University of Chinese Medicine, Guangdong Second Hospital of Traditional Chinese Medicine, Department of Rheumatism, Guangzhou, PR China; National Institutes of Health, UNITED STATES OF AMERICA

## Abstract

**Objective:**

The study aimed to examine the association between obesity and hyperuricemia in *ob/ob* mice.

**Methods:**

An animal model of obesity was developed using male ob/ob mice. Biochemical parameter test kits were used to measure serum uric acid (UA), hepatic xanthine oxidase (XOD) activity, serum creatinine (Scr), serum lipid profiles, and blood urea nitrogen (BUN). Then, liver tissues were collected for hematoxylin and eosin (H&E) staining, flow cytometry, and western blot (WB) analysis. Furthermore, Huh-7 cells were co-cultured with THP-1 macrophages for 24 hours, with or without LPS + IFN-γ or PA, and subsequently analyzed for XOD activity. In addition, the Huh-7 cells stimulated with PA were analyzed by metabolomics and validated by WB and RT-qPCR.

**Results:**

Levels of Serum lipid profiles, UA, and XOD activity are elevated in *ob/ob* mice. In *ob/ob* mice, liver M1 macrophage polarization is markedly enhanced. In vitro studies show that elevated XOD activity in hepatocytes during hyperlipidemia does not correlate with M1 macrophage polarization. Metabolomics showed that the XOD activity of hepatocytes in hyperlipidemia may be related to pyruvate metabolism. Moreover, the protein and mRNA levels of pyruvate dehydrogenase (PDH), an enzyme that limits pyruvate accumulation, were significantly down-regulated in Huh-7 cells with PA stimulation.

**Conclusion:**

Hyperuricemia in ob/ob mice relates to hepatocellular pyruvate metabolism/ xanthine oxidase axis.

## 1. Introduction

Obesity, characterized by excessive adipose tissue, affects 13% of the global adult population and is regarded as an epidemic [1]. It significantly elevates the likelihood of metabolic dysfunctions, including nonalcoholic fatty liver disease, type 2 diabetes, and so on [2]. Hyperuricemia (HUA), a purine metabolic disorder affected by genetic and environmental elements, is recognized as the fourth hyper-disease following hypertension, hyperglycemia, and hyperlipidemia. HUA is an established risk factor for conditions including gout, renal dysfunction, arthritis, cardiovascular diseases, and metabolic disorders [3–5]. According to the global report, around 41 million individuals globally suffer from gout due to elevated serum uric acid (SUA) levels, posing significant public health challenges and increasing attention [6,7].

Various studies have shown the association between hyperuricemia and obesity, which share a complex comorbid relationship [8–10]. A meta-analysis revealed a progressive association between elevated SUA levels and increased prevalence of dyslipidemia [11]. Another study found that fenofibrate supplementation lowered uric acid levels in gout patients on allopurinol or febuxostat, without significantly affecting renal or hepatic functions [12]. At the same time, clinical studies have indicated associations between obesity and hyperuricemia; however, the mechanism of increased risk of hyperuricemia due to obesity is not fully understood.

XOD is an essential enzyme in UA synthesis, converting hypoxanthine to UA. A notable increase in hepatic XOD activity is observed in both high-fat diet-induced mice and *ob/ob* mice. Interestingly, our findings indicate that XOD protein levels were similar in both WT and ob/ob livers; however, XOD activity was significantly higher in ob/ob mice compared to WT mice. In the present study, the mechanisms of elevated XOD activity in obesity were investigated in *vitro* and in *vivo*.

## 2. Methods

### 2.1 Animals

Ten-week-old male C57BL/6 mice and leptin-deficient male ob/ob mice were housed by Viton Lihua Laboratory Animal Technology Co. (Guangdong, China, SCXK, 2022−0063) and raised at the Animal Experimental Center of Shenzhen Zhongxun Precision Medicine Research Institute (Guangdong, China, SCXK, 2019–0210) within a specific pathogen-free environment, maintained at 20–26 °C with 40–70% relative humidity. Experiments were carried out after 7 days of adaptive feeding. The experimental protocols were approved by the Animal Experiment Ethics Committee of the Shenzhen Zhongxun Precision Medicine Research Institute (ZXJZ202206010015). Subsequently, all experimental animals were humanely euthanized using isoflurane anesthesia followed by neck breaking in accordance with American Veterinary Medical Association guidelines to minimize the suffering experienced by the mice.

### 2.2 Cell culture

THP-1 and Huh7 cells are sourced from the Cell Bank of the Chinese Academy of Sciences in Shanghai, China. Cells were cultured in DMEM (C111995500BT, Gibco, USA) supplemented with 10% FBS (164210, Priscilla, China) at 37°C and 5% CO2. THP-1 cells were differentiated into macrophages for 48 hours using phorbol-12-myristate-13-acetate (PMA; S1819, Beyotime, China, 10 ng/mL).

For co-culture studies, THP-1 macrophages and Huh7 cells were seeded at a ratio of 1:2 (10^6^ THP-1 macrophages and 2 × 10^6^ Huh7 cells per well) into Transwell co-culture chambers (3452, Corning, USA) with a 0.4 µm pore size. THP-1 macrophages were seeded in the upper insert, while Huh7 cells were seeded in the lower compartment. After 24 hours of co-culture in complete DMEM, the medium was replaced with fresh DMEM containing 0.2 mM palmitic acid (PA; SLCH6263, Sigma-Aldrich, USA), which had been conjugated with 10% fatty acid–free BSA (ST025, Beyotime, China) to enhance cellular uptake. The final PA stock solution was prepared at 400 mM in BSA. In some groups, lipopolysaccharide (LPS; L280, Sigma-Aldrich, 100 ng/μL) and interferon-γ (IFN-γ; 300-02-100, PEPROTECH, 20 ng/μL) were added instead of PA to stimulate macrophage activation. Co-cultures were maintained for 24 h following treatment [13], after which hepatocytes were harvested for downstream assays, including XOD activity measurement.

### 2.3 XOD activity measurement

The XOD activity in tissues and cells was measured using the XOD activity assay kit from Solarbio (BC1095). For cells, 5 million cells were collected in a centrifuge tube, centrifuged, and the supernatant was discarded. Then, the lysis buffer and detection solution were added. Use ultrasonication to split cells (place on ice, ultrasonic power 200W, working time 3 seconds, interval 10 seconds, repeat for 30 times). After centrifugation at 800 g, 4 °C for 10 min, the supernatant was collected on ice, and the absorbance at 530 nm was immediately recorded at 20 min, respectively, and the final results were calculated. The results were quantitatively analyzed using a cell counting method. For animal tissues, a certain amount of tissue was weighed and added to the lysis buffer and detection solution. The tissue was homogenized thoroughly using a homogenizer or mortar and pestle on ice. After centrifugation at 800 g, 4 °C for 10 min, the supernatant was collected on ice, and the absorbance at 530 nm was immediately recorded at 20 min, respectively, and the final results were calculated. The protein concentration was quantitatively analyzed using the BCA method. The assay was validated before experimental use by running known positive and negative controls to confirm assay specificity and dynamic range.

### 2.4 Biochemical analysis

Serum levels of UA, Scr, BUN, TC, HDL-C, TG, and LDL-C were measured using commercially available kits (Jiancheng, Nanjing, China) according to the manufacturer’s instructions.

### 2.5 Histopathology

Mouse livers were fixed in 4% paraformaldehyde at 4°C overnight and embedded in paraffin, then sectioned into 3 mm slices. Sections underwent hematoxylin and eosin (H&E) staining. Images were acquired and analyzed using an Axio Imager M2 microscope (Carl Zeiss, Germany).

For additional assessment of hepatic fat deposition, frozen liver sections were stained with Oil Red O (G1015, Servicebio) and counterstained with hematoxylin. Stained sections were visualized and imaged using the Axio Imager M2 microscope (Carl Zeiss, Germany).

### 2.6 Western blotting

Cells and livers were harvested for lysis in pierce^TM^ RIPA buffer (89900, Thermo scientific, USA), which included proteinase/phosphatase inhibitors (P1050, Beyotime, China). A BCA kit (P0012, Beyotime, China) was employed to measure the protein content. For each lane, 20–30 μg of total protein lysate was loaded for SDS-PAGE. Proteins were separated using 10% SDS-PAGE and then transferred to PVDF membranes (Millipore, Billerica, MA, USA). The membranes were blocked with TBST solution containing 5% non-fat milk at room temperature for 1 hour, then incubated overnight at 4 °C with primary antibodies: β-actin (#4970, CST, USA), CD86(13395–1-AP, Proteintech, China), Interleukin (IL)-1β (ab254360, Abcam, USA), Nod-like receptor protein 3 (NLRP3) (#15101, CST, USA), CD206(#24595, CST, USA), XOD(ab109235, Abcam, USA), CD80 (66406–1-Ig, Proteintech, China) and pyruvate dehydrogenase (PDH) (#2784, CST, USA). This was followed by a 1-hour incubation with secondary antibodies (#7074, #7076, CST, USA). The ECL reagent (Bio-Rad, Hercules, CA, USA) was used to observe the protein bands. ImageJ software was used to analyze band intensities.

### 2.7 Flow cytometry

Livers were homogenized and passed through a 70 μm filter (352,340, Corning, USA) to create single-cell suspensions in PBS with 1% FBS. Red cell depletion was carried out on the single-cell suspensions, and the resultant cells were collected for subsequent staining. Subsequently, cells were stained for 20 min at 4 °C with Zombie Violet (423113, Biolegend, USA), PE/Cyanine7 anti-mouse/human CD11b (101216, Biolegend), APC/Cyanine7 anti-mouse CD45 (157618, Biolegend), FITC anti-mouse F4/80 (123107, Biolegend), and PE anti-mouse CD86 (159203, Biolegend). Subsequently, zombie violet (423113, Biolegend, USA), PE/cyanine7 anti-mouse/human CD11b (101216, Biolegend, USA), APC/cyanine7 anti-mouse CD45 (157618, Biolegend, USA), FITC anti-mouse F4/80 (123107, Biolegend, USA) and FITC anti-mouse F4/80 (123107, Biolegend, USA) were used to culture cells Pe/cyanine7 anti-mouse/human CD11b (101216, Biolegend, USA), PE anti-mouse CD86 antibody (159203, Biolegend, USA) and FITC anti-mouse F4/80 (123107, biolegend, USA), 20 minutes at 4 ˚C. After immobilization and permeabilization of the cells using the immobilization/permeabilization Kit (554714, BD, USA), the cells were subjected to incubation with APC anti mouse CD206 antibody (141708, Biolegend, USA) at 4 °C for 20 minutes. Lastly, the samples were detected by flow cytometry (FACScanto II, BD, USA).

### 2.8 Metabolomics

Huh-7 cells were treated with either PA-BSA (0.2 mM) or BSA for 24 hours. Proteins and metabolites were extracted using cold methanol/acetonitrile (1:1, v/v). Centrifuge the mixture at 14,000 g for 20 minutes to obtain the supernatant, which was then dried using a vacuum centrifuge. The sample was re-dissolved in 100 μl of a 1:1 acetonitrile/water solution, centrifuged at 14,000 g at 4 °C for 15 minutes, and the supernatant was analyzed using ultra-performance liquid chromatography (1290 Infinity LC, Agilent Technology, USA) and a quadrupole time-of-flight analyzer (AB TripleTOF 6600, AB SCIEX, USA). MetaboAnalyst was utilized for pathway analysis to enhance interpretation.

The raw MS data were converted to MzXML files using ProteoWizard MSConvert before importing into freely available XCMS software. For peak picking, the following parameters were used:centWave m/z = 10 ppm, peak width = c (10, 60), prefilter = c (10, 100). For peak grouping, bw = 5, mzwid = 0.025, minfrac = 0.5 were used. CAMERA (Collection of Algorithms of MEtabolite pRofile Annotation) was used for the annotation of isotopes and adducts. In the extracted ion features, only the variables having more than 50% of the nonzero measurement values in at least one group were kept. Compound identification of metabolites was performed by comparing the accurate m/z value (< 10 ppm) and MS/MS spectra with an in-house database established with available authentic standards.

### 2.9 Quantitative real-time PCR (qRT-PCR)

Cellular RNA was extracted using the RNA Pure Kit (G3640, Servicebio), and cDNA synthesis was performed with SweScript All-in-One RT SuperMix and gDNA remover (G3337, Servicebio). qRT-PCR was performed using SYBR Green qPCR Master Mix (G3326, Servicebio, China) on the Applied Biosystems QuantStudio 5 platform (Thermo Fisher Scientific, USA). Refer to Table 1 for primer details. Data were quantified using the 2^−△△Ct^ method.

**Table 1:** Primer sequences for qRT-PCR.

Gene symbol	Forward 5’-3’	Reverse 5’-3’
Human		
PDK	AGGGCATCATTCACAGGGAC	CAAGAGCCCAAAGGTCTGAACT
MPC1	TGCCCTCTGTTGCTATTCTTTGA	TGGGCTACTTCATTTGTTGCGT
MPC2	CCTCGATAAAGTGGAGCTGATG	TTGACCAAATAAACCCTGTAGCC
PKM	CACCTGTACCGTGGCATCTTC	AACATTCATGGCAAAGTTCACCC
PDH	CGAATTGGAATCCCAGTCAGAAG	AGTTGAGTTGGTGCTGGCATG
ENO1	CCAGTGCAGGAATCCAGGTAG	CTCGGTCACGGAGCCAATCT
β-actin	CACCCAGCACAATGAAGATCAAGAT	CCAGTTTTTAAATCCTGAGTCAAGC

### 2.10 Molecular docking

Obtain protein information from the Uniprot website (https://www.uniprot.org/uniprotkb) and protein structures from the SCSB PDB database (https://www.rcsb.org/). Import the obtained structures into Discovery Studio 2019 for protein structure optimisation. This process mainly includes dehydration, hydrogenation, charge completion, amino acid completion, side chain completion, etc. Finally, the optimised protein structure is obtained, and the protein structure is exported as a PDB file. Obtain small molecule structures from the PubChem website and perform energy minimisation using Discovery Studio 2019, saving the results as a PDB file. Prepare protein and small molecule PDBQT files using Autodock 1.5.6 and perform docking using Autodock Vina 1.2.6. Finally, perform visualisation analysis using PyMOL 3.1 and Discovery Studio 2019.

### 2.11 Statistical analysis

The findings were presented as the means ±SEMs. Statistical analyses were performed with SPSS 25.0. Multiple groups were compared by one-way ANOVA plus the LSD test or Dunnett’s T3 multiple comparison test. *p* < 0.05 was considered statistically significant.

## 3. Results

### 3.1. Levels of uric acid (UA) and xanthine oxidase (XOD) activity are elevated in ob/ob mice

**Fig 1:**
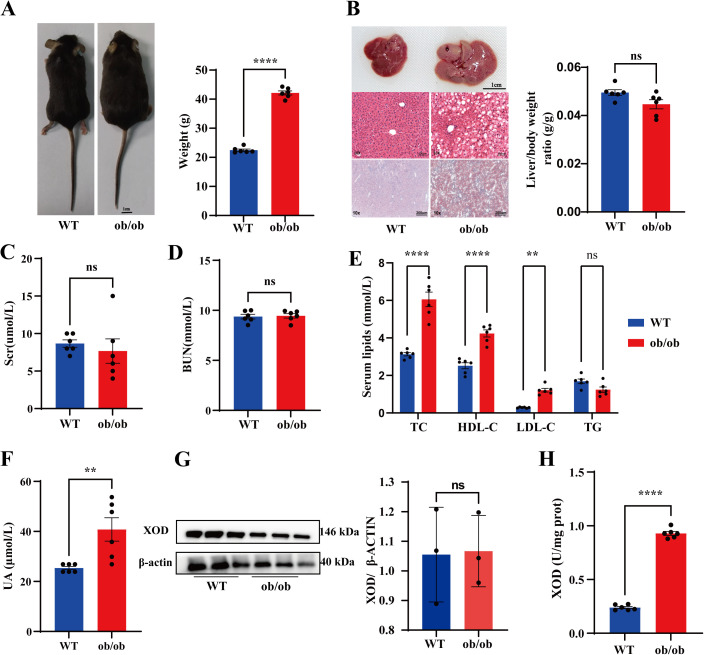
Levels of uric acid (UA) and xanthine oxidase (XOD) activity are elevated in ob/ob mice. **(A)** Representative photographs (scale bars, 1 cm) and body weights of wild-type (WT) and ob/ob mice. **(B)** Representative images of livers (scale bars, 1 cm), H&E-stained, and Oil red O–O-stained liver sections (scale bars, 200 µm) from WT and ob/ob mice. The ratio of liver weight to body weight of mice. **(C)** Serum creatinine (Scr) levels. **(D)** Blood urea nitrogen (BUN) levels. **(E)** Serum lipid profiles: total cholesterol (TC), total triglyceride (TG), high-density lipoprotein cholesterol (HDL-C), and low-density lipoprotein cholesterol (LDL-C) in WT and ob/ob mice. **(F)** Serum uric acid levels. **(G)** Western blotting for XOD in the liver of WT and ob/ob mice. **(H)** Hepatic xanthine oxidase (XOD) activity. Data are presented as mean ± SEM for each group (n = 3-6). *p <0.05, **p < 0.01, ****p < 0.0001, n.s. not significant.

We investigated the association between obesity and hyperuricemia by analyzing relevant indicators in 10-week-old male wild-type (WT) and obese ob/ob mice. As expected, ob/ob mice showed a significantly higher body weight than WT mice (Fig 1A). The liver in ob/ob mice was notably larger than in WT mice. Body weight and liver ratio decreased slightly, but the change was not statistically significant.H&E staining indicated notable hepatic steatosis in ob/ob mice, marked by enhanced fat deposition and enlarged fat vacuoles (Fig 1B). Oil Red O staining confirmed increased lipid accumulation in hepatocytes of ob/ob mice compared to WT controls, consistent with H&E observations (Fig 1B). There was no statistically significant difference in renal function, as indicated by serum creatinine (Scr) and blood urea nitrogen (BUN) levels, between ob/ob mice and WT mice (Fig 1C and 1D). Ob/ob mice exhibited dyslipidemia, characterized by significantly elevated serum levels of total cholesterol (TC), high-density lipoprotein cholesterol (HDL-C), and low-density lipoprotein cholesterol (LDL-C). Total triglyceride (TG) levels showed a slight decrease compared to WT mice, but this reduction was not statistically significant (Fig 1E). Serum UA levels were higher in ob/ob mice compared to WT (Fig 1F). Given the pivotal role of hepatocyte XOD in systemic UA homeostasis [14], we assessed XOD protein expression. Western blot analysis showed no difference in XOD protein levels between WT and ob/ob livers (Fig 1G). XOD activity was significantly higher in ob/ob mice compared to WT mice (Fig 1H). The results suggest elevated UA levels and XOD activity in *ob/ob* mice.

### 3.2. M1 macrophage polarization is significantly promoted in the liver of ob/ob mice

**Fig 2:**
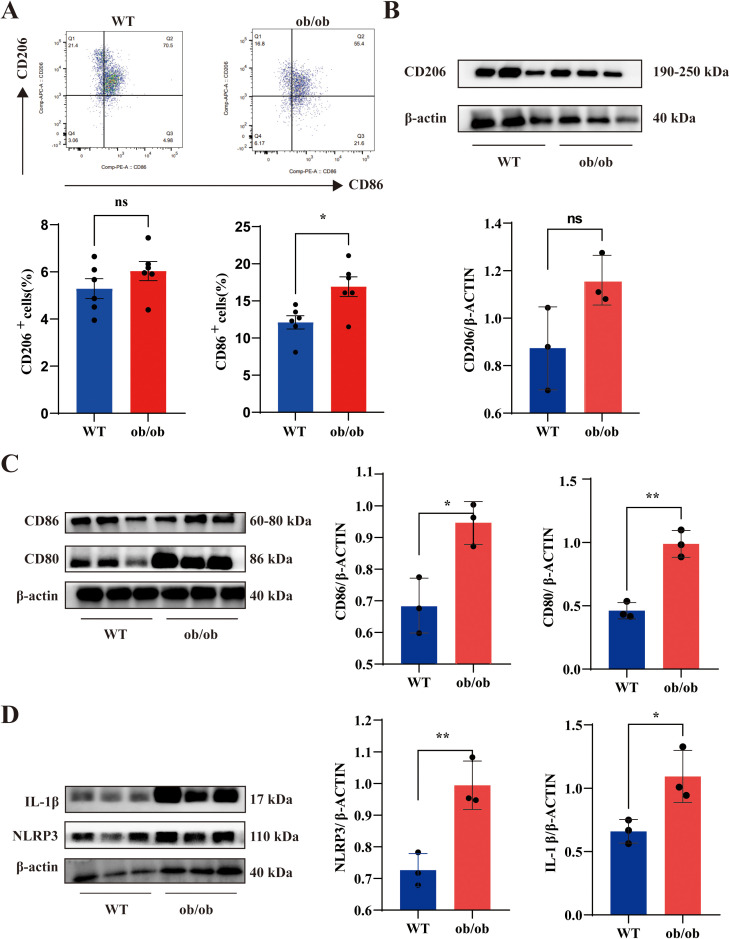
M1 macrophage polarization is significantly promoted in the liver of ob/ob mice. **(A)** Through flow cytometry, the percentages of M1 macrophages (F4/80 + CD11b + CD86 + cells) and M2 macrophages (F4/80 + CD11b + CD206 + cells) in the livers of WT and ob/ob mice were determined. **(B-D)** Western blotting was employed to analyze CD206, CD86, CD80, NLRP3, and IL-1β in the liver of WT and ob/ob mice. The data are exhibited as the mean ± SEM in each group (n = 3-6).*p < 0.05, **p < 0.01, n.s. not significant.

Previous studies have shown that obesity leads to macrophage polarization [15]. At the same time, hyperuricemia is closely related to macrophage polarization [16,17]. Therefore, we investigated the role of macrophages in ob/ob mice by flow cytometry of the livers. Flow cytometry analysis showed a significant increase in F4/80 + CD11b+CD86 + M1-like macrophages, while F4/80 + CD11b+CD206 + M2-like macrophages exhibited a modest, non-significant increase (Fig 2A). In addition, compared with WT mice, the expression of CD80 and CD86 in the liver of ob/ob mice was significantly higher than in WT mice, whereas CD206 expression showed no significant change (Fig 2B and 2C). NLRP3 and IL-1β protein levels were notably elevated in obese mice compared to WT mice (Fig 2D). These findings collectively verify the hepatic M1 polarization and NLRP3-mediated inflammation in *ob/ob* mice.

### 3.3. Increased XOD activity of hepatocytes in hyperlipidemia is not associated with M1 macrophage polarization in vitro

The results described above suggested that M1 macrophage polarization due to a high-fat environment may affect XOD activity, leading to elevated levels of UA. We first incubated THP-1 macrophages with 0.2 mM PA to mimic high fatty acid exposure for 24 hours, and we examined NLRP3 and CD80 protein expression at 0, 4, 6, 8, 10, 12, and 24 h after PA stimulation.

**Fig 3:**
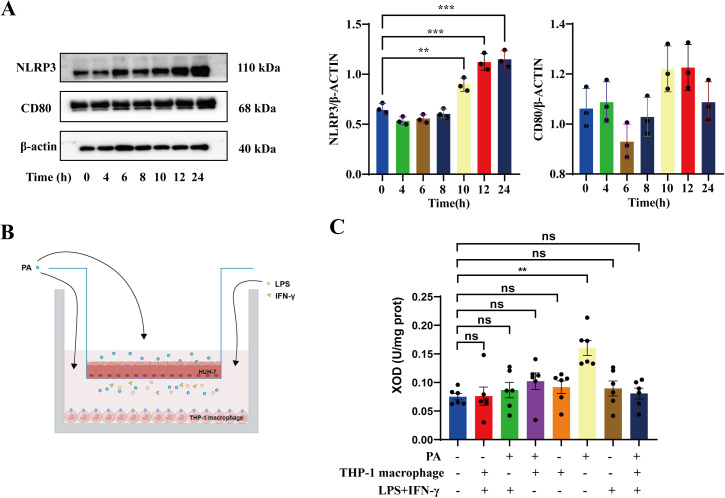
Increased XOD activity of hepatocytes in hyperlipidemia is not associated with M1 macrophage polarization in vitro. **(A)** Immunoblot analysis of NLRP3, CD80 in THP-1 macrophages followed by palmitic acid (PA) treatment for 0–24 hours. **(B)** Schematic showing that HUH-7 cells were co-cultured with THP-1 macrophages with or without LPS + IFN-γ or PA. **(C)** Hepatic XOD activity in Huh-7 cells. The data are exhibited as the mean ± SEM in each group (n = 3-6). *p < 0.05, **p < 0.01, ***p < 0.001, n.s. not significant.

To evaluate the activation status of macrophages in response to different stimuli, we examined the expression of CD80 and CD206, which are well-established markers of M1 and M2 macrophage polarization, respectively. CD80 is a co-stimulatory molecule typically upregulated in pro-inflammatory (M1) macrophages, whereas CD206 (mannose receptor) is commonly used as a marker for alternatively activated (M2) macrophages [18,19]. These markers provided a framework for assessing the immunophenotypic polarization of THP-1-derived macrophages under the influence of palmitic acid and LPS/IFN-γ stimulation. NLRP3 protein level increased in a time-dependent manner following PA treatment, while CD80 expression remained stable throughout the 24-h stimulation period (Fig 3A). These trends were validated through densitometric analysis, confirming the selective induction of NLRP3 without accompanying upregulation of CD80. Thus, PA stimulation alone did not promote M1 macrophage polarization. Then, we co-cultured HUH-7 cells and THP-1 macrophages with or without LPS + IFN-γ or PA and determined the hepatic XOD activity (Fig 3B). Interestingly, the XOD activity was significantly higher in PA stimulation alone than in any other circumstances (Fig 3C). These data suggest that the elevated xanthine oxidase activity isn’t attributable to M1 macrophage polarization.

### 3.4. Increased XOD activity of hepatocytes in hyperlipidemia may be related to pyruvate metabolism

**Fig 4:**
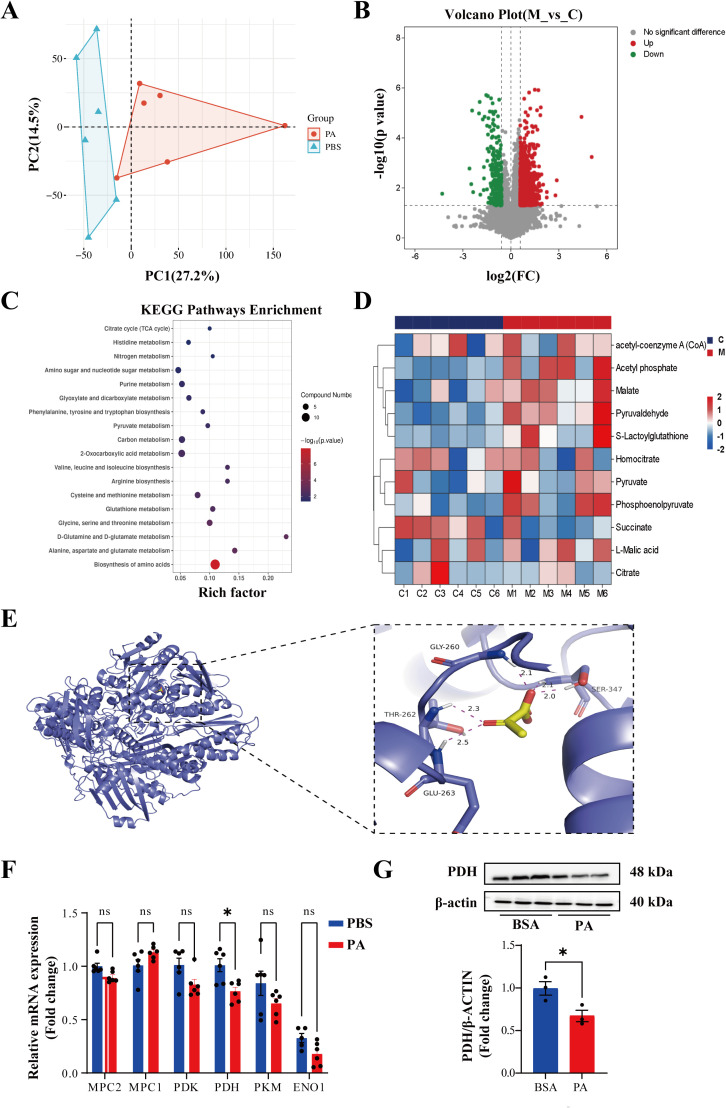
Increased XOD activity of hepatocytes in hyperlipidemia may be related to pyruvate metabolism. **(A)** Principal component analysis (PCA) based on metabolomics of HUH-7 cells with PA or without PA. **(B)** The volcano plot shows the differential gene expressions as blue and red dots. **(C)** KEGG pathway enrichment analysis based on metabolomics data. **(D)** Heatmap of differential metabolites related to tricarboxylic acid (TCA) cycle and pyruvate metabolism in HUH-7 cells with PA or not. **(E)** Molecular docking of XOD and pyruvate. **(F)** Quantitative RT-PCR analysis of genes related to pyruvate metabolism. **(G)** Western blot analysis of PDH protein expression and quantification. The data are exhibited as the mean ± SEM in each group (n = 3-6). *p < 0.05, **p < 0.01, n.s. not significant.

To further understand how the high-fat environment affects hepatocellular XOD activity, we next conducted a metabolomics analysis on HUH-7 cells with PA stimulation. Through principal component analysis (PCA) , we can observe that there are significant differences between PA-treated or not Huh-7 cells (Fig 4A). In the PA group, 335 metabolites were down-regulated and 1524 metabolites were up-regulated compared to the control group (Fig 4B). KEGG analysis showed that pathways included tricarboxylic acid (TCA) cycle, pyruvate metabolism and so on (Fig 4C). A heatmap (Fig 4D) illustrates the differential metabolites associated with the TCA cycle and pyruvate metabolism in HUH-7 cells, both with and without PA (Fig 4D). We used molecular docking technology to further explore the relationship between pyruvate and XOD. The relative energy of pyruvate-XOD was −4.7 kcal/mol, and the binding affinity between pyruvate and XOD was high, suggesting a good binding capacity. The ligand forms hydrogen bonds with GLU263, THR262, GLY260, and SER347 on the protein to form stable interactions. (Fig 4E). Afterward, qPCR analysis was performed to investigate the changes in genes involved in the pyruvate metabolism. The study found a significant down-regulation of PDH mRNA in HUH-7 cells following PA stimulation (Fig 4F). Western blotting analysis showed a similar result of PDH protein expression (Fig 4G). These results indicate that increased XOD activity of hepatocytes in ob/ob may be related to pyruvate metabolism.

## 4. Discussion

HUA constitutes a metabolic disorder marked by elevated levels of UA in the bloodstream, being the primary outcome of purine metabolism [20]. HUA shares a complex comorbid relationship with obesity [21]. In this study, we found that serum uric acid (UA) levels were higher in ob/ob mice compared to wild-type (WT) mice. Indeed, HUA is mainly caused by hepatic UA overproduction and reduced renal excretion. It demonstrated that there is no statistical difference in renal function (Scr and BUN levels) between ob/ob mice and WT mice. It indicated that the mechanism of hyperuricemia in ob/ob mice may not be related to renal excretion. Strikingly, XOD, expressed in the liver, is an essential enzyme in the metabolism of hypoxanthine and xanthine for UA production [22]. It catalyzes the final step of purine catabolism, converting hypoxanthine to xanthine, then to UA, and producing reactive oxygen species (ROS) [14,23]. Several studies have shown that hepatic XOD activity was increased in HFD-induced mice [24,25]. Similarly, XOD activity was significantly higher in ob/ob mice compared to WT mice. It suggests that hyperuricemia in ob/ob mice may be associated with abnormal hepatic xanthine oxidase (XOD) activity.

Former studies indicate that a high-fat diet enhances macrophage polarization and XOD activity [26]. Macrophages differentiate into distinct functional phenotypes, specifically classical M1 and alternative M2 macrophages [27]. In obese mice, proinflammatory M1 macrophages accumulate and are linked to insulin resistance, whereas anti-inflammatory M2 macrophages are largely absent [28]. Flow cytometry and WB results suggested that M1 macrophage polarization is significantly promoted in the ob/ob mice. To further investigate the effect of macrophage polarization on hepatic XOD activity, Huh-7 cells were co-cultured with THP-1 macrophages with or without LPS + IFN-γ or PA in *vitro*. Unexpectedly, M1 macrophages did not increase the XOD activity in Huh-7 cells. Thus, this study uncovers that increased hepatic XOD activity in ob/ob mice is not associated with M1 macrophage polarization.

Notably, the XOD activity was significantly higher in PA stimulation alone. KEGG analysis of metabolomics indicated that the pyruvate metabolism pathway was enriched in PA-stimulated Huh-7 cells. Indeed, circulating pyruvate concentrations are increased in NAFLD patients [29]. Pyruvate plays a key role in central carbon metabolism. The role of pyruvate metabolic disorders is particularly prominent in neurodegeneration, cancer, heart failure, and other diseases [30]. We used molecular docking technology to further explore the relationship between pyruvate and XOD. The relative energy of pyruvate-XOD was −4.7 kcal/mol, and the binding affinity between pyruvate and XOD was high, suggesting a good binding capacity. The metabolism of pyruvate is related to enzymes such as MPC and PDH, and it has been detected that PDH is significantly altered, while other enzymes are not significantly altered. The mitochondrial PDH complex (PDC) serves as the gatekeeper and rate-limiting enzyme in mitochondrial glucose oxidation, facilitating the decarboxylation of pyruvate to produce acetyl-coenzyme A (CoA) [31]. PDH is essential for cellular energy metabolism and biosynthetic pathways. Many studies have shown that xanthine oxidase regulates PDH expression [32]. Hyperuricemia in ob/ob mice may be related to hepatocellular pyruvate metabolism/xanthine oxidase axis.

Although our findings suggest a potential link between pyruvate metabolism and XOD activity, particularly implicating PDH levels, the current data are correlative and do not establish a definitive mechanistic relationship. Research has shown that the intervention and regulation of PDH activity has become an important avenue of research for the treatment of various metabolic disorders [33]. Furthermore, many reports indicate that ROS produced by xanthine oxidase can inhibit the activity of PDH [34]. Therefore, reduced PDH activity may have a negative feedback effect on XOD.

Our findings demonstrate increased XOD activity and altered PDH expression in ob/ob mice; it remains unclear whether PDH downregulation is causative or merely correlative in the context of hyperuricemia. Given the complexity of hepatic metabolic regulation, it is plausible that other upstream pathways or regulatory proteins, such as AMPK [35] and Protein phosphatase 2A [36], may contribute to XOD activation either directly or indirectly via modulation of cellular redox state or mitochondrial dysfunction. Moreover, inflammatory cytokines induced by M1 macrophage polarization may independently stimulate hepatic XOD activity through signaling cascades, such as JAK/STAT or NF-κB [37]. These possibilities suggest that the observed PDH suppression may reflect broader metabolic reprogramming in obese livers rather than a direct driver of uric acid elevation.

**Fig 5:**
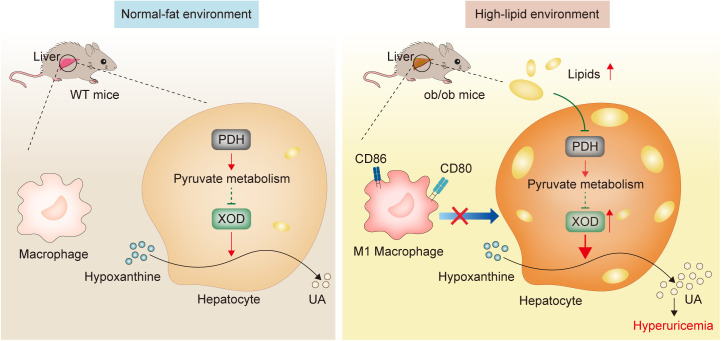
Schematic diagram of the mechanism of the regulation of XOD activity in ob/ob mice and WT mice.

The primary strength of our study lies in offering an initial explanation of the link between obesity and HUA in ob/ob mice (Fig 5). The major limitation of the present study was that the underlying mechanism by which pyruvate metabolism/PDH modulates HUA in ob/ob mice has not been clarified. Further mechanistic validation, such as modulation of PDH protein levels using siRNA or CRISPR-based approaches or utilizing PDH inhibitors in a cell-culture-based system, and assessing its impact on XOD activity, will be performed in the future. Secondly, we have recognized the choice of the ob/ob mouse animal model as a constraint of this research. Future investigations ought to further examine obesity in other paradigms, like high-fat diet-induced obesity, to offer a more all-encompassing comprehension. Thirdly, although Huh7 cells were employed in this study to investigate the hepatic mechanisms underlying uric acid metabolism, it is acknowledged that this hepatocellular carcinoma-derived line does not fully recapitulate the functional phenotype of primary hepatocytes. Nevertheless, the use of Huh7 cells enabled efficient genetic and metabolic manipulations to delineate key mechanistic pathways. Future studies involving targeted modulation of PDH expression *in vivo*, such as through adeno-associated virus (AAV)-mediated hepatocyte-specific overexpression or knockdown, are warranted to further validate the causal role of PDH in regulating hepatic uric acid production and hyperuricemia.

## Supporting information

S1 File Original Western blot. (PDF)

S2 File HE and Oil red staining. (PDF)

S3 Data Data for figures. (XLSX)
