# Supplementary figures and images for: Hyperuricemia in ob/ob mice relates to hepatocellular pyruvate metabolism/ xanthine oxidase axis
Source: PLoS One. 2025 Aug 6;20(8):e0328794. doi: 10.1371/journal.pone.0328794 (PMC12327685; doi:10.1371/journal.pone.0328794)

Fig1G--XOD

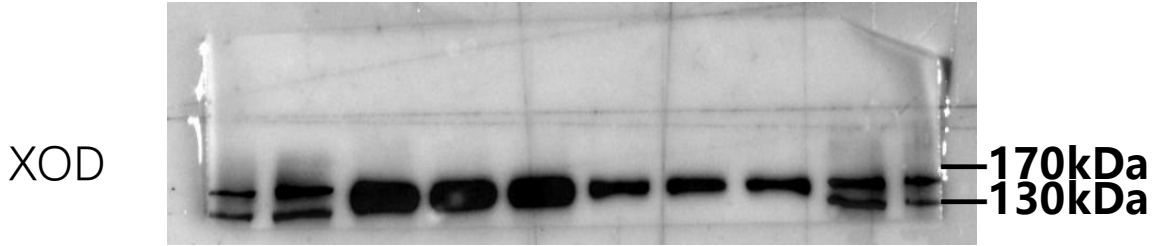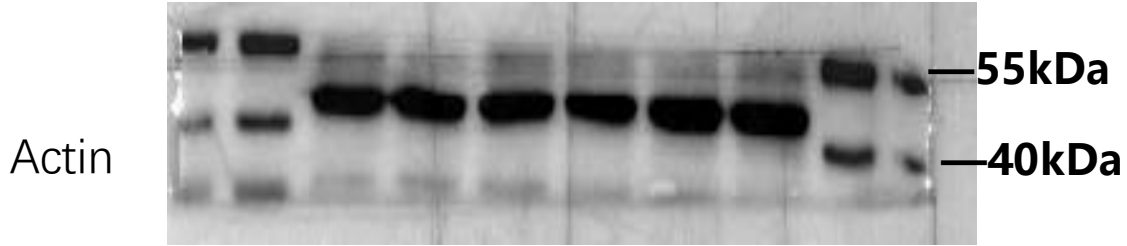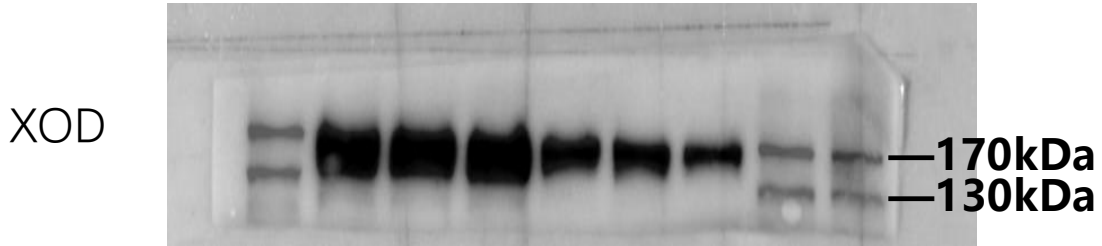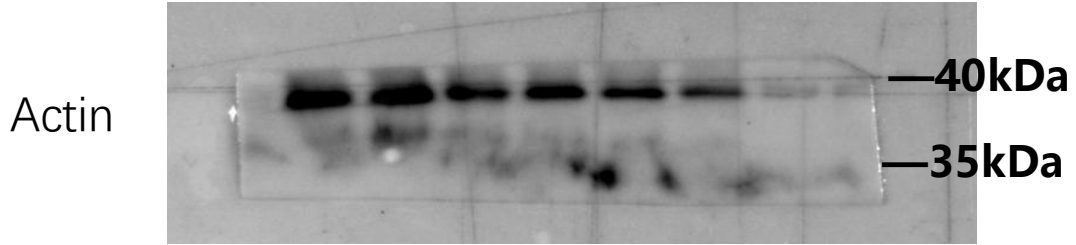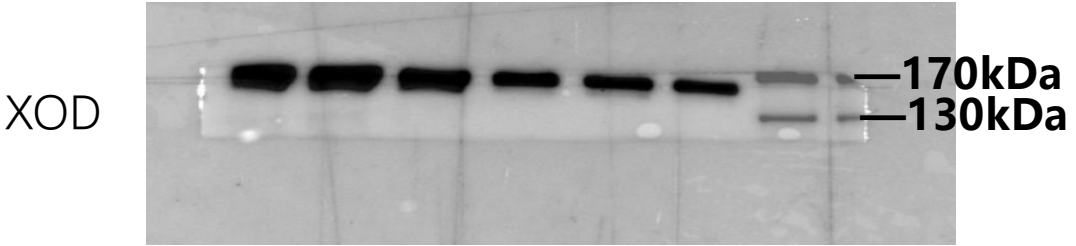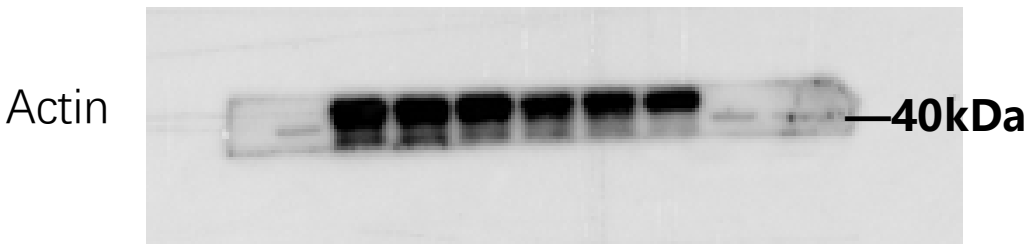

Fig2.B--CD206

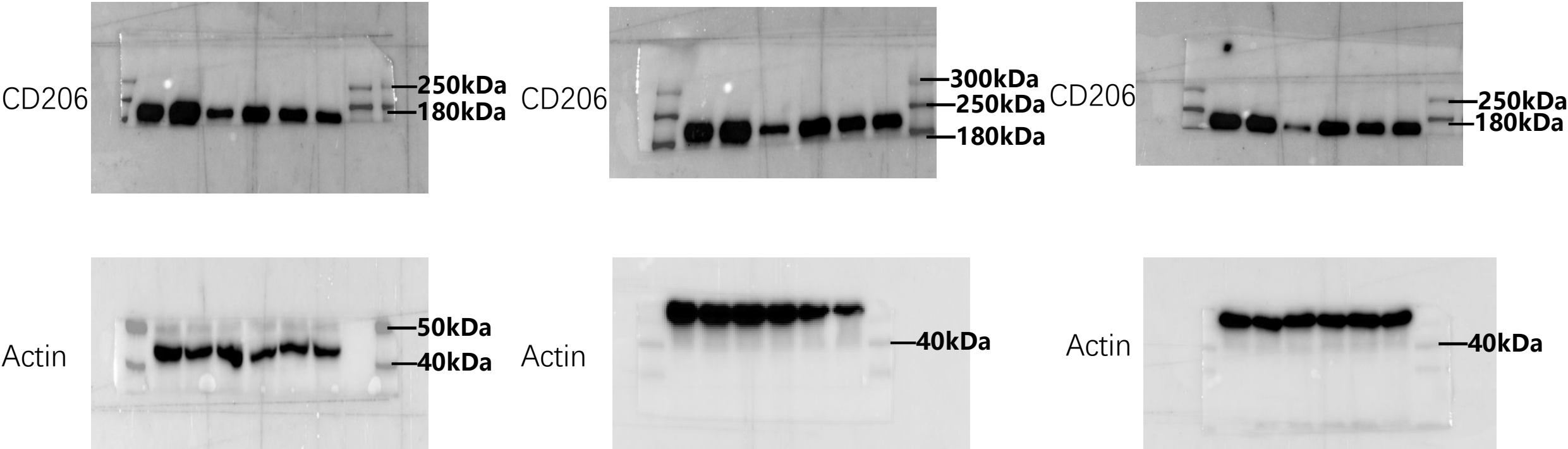

Fig2.C--CD86、CD80

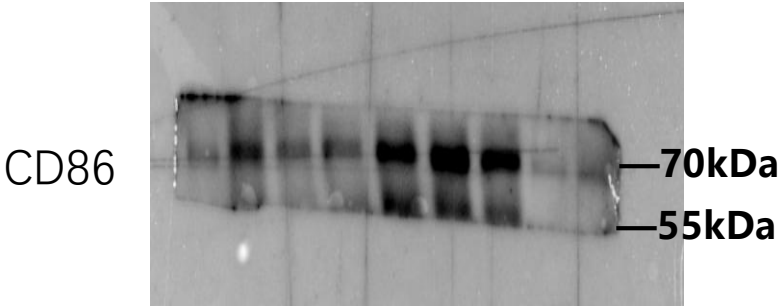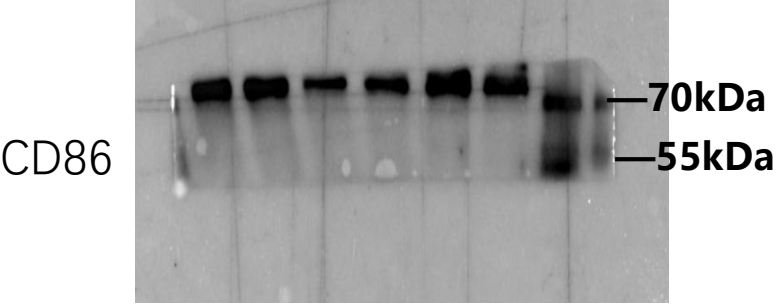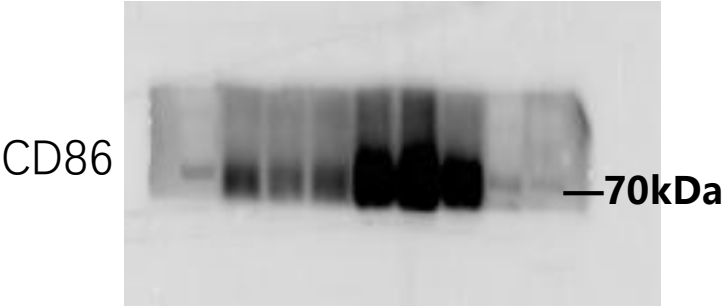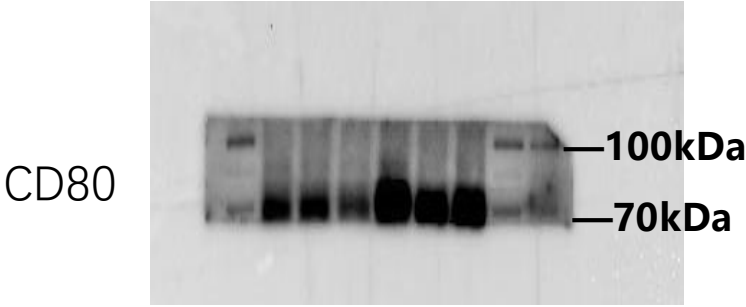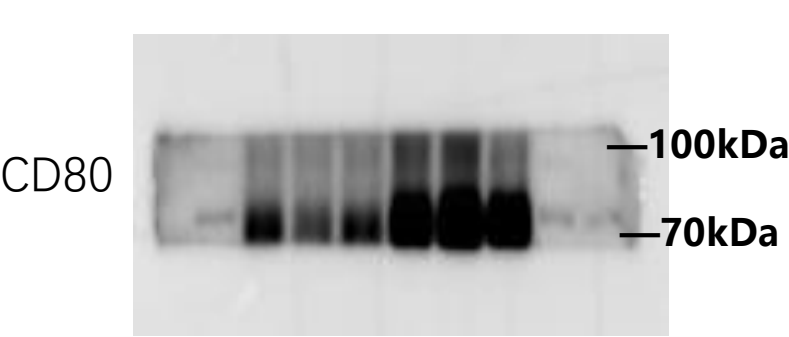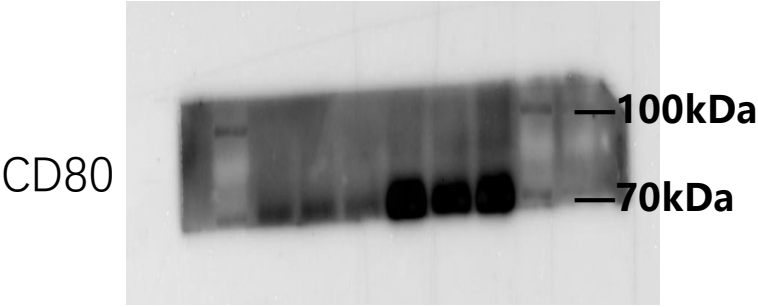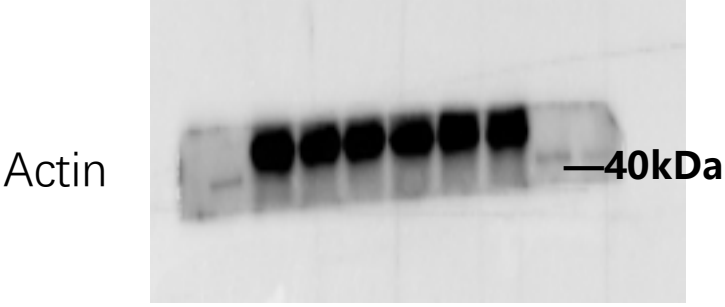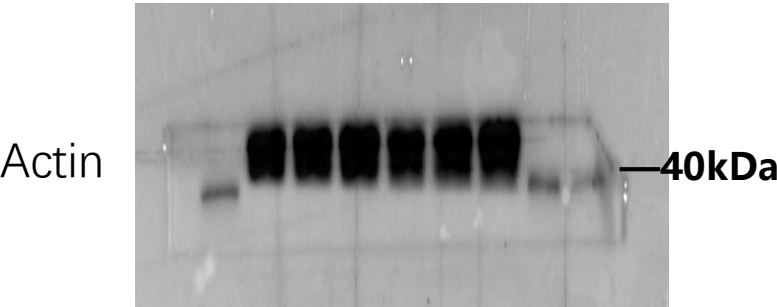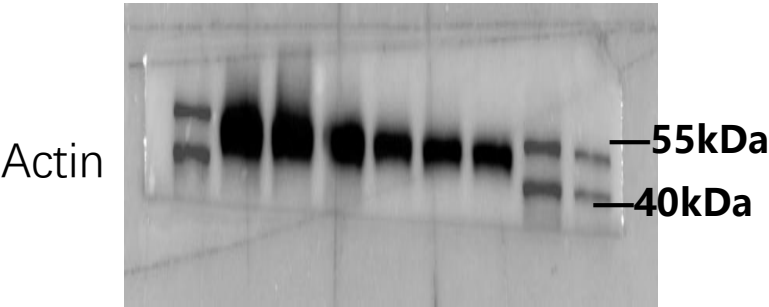

Fig2.D--IL-1 $\beta$ 、NLRP3

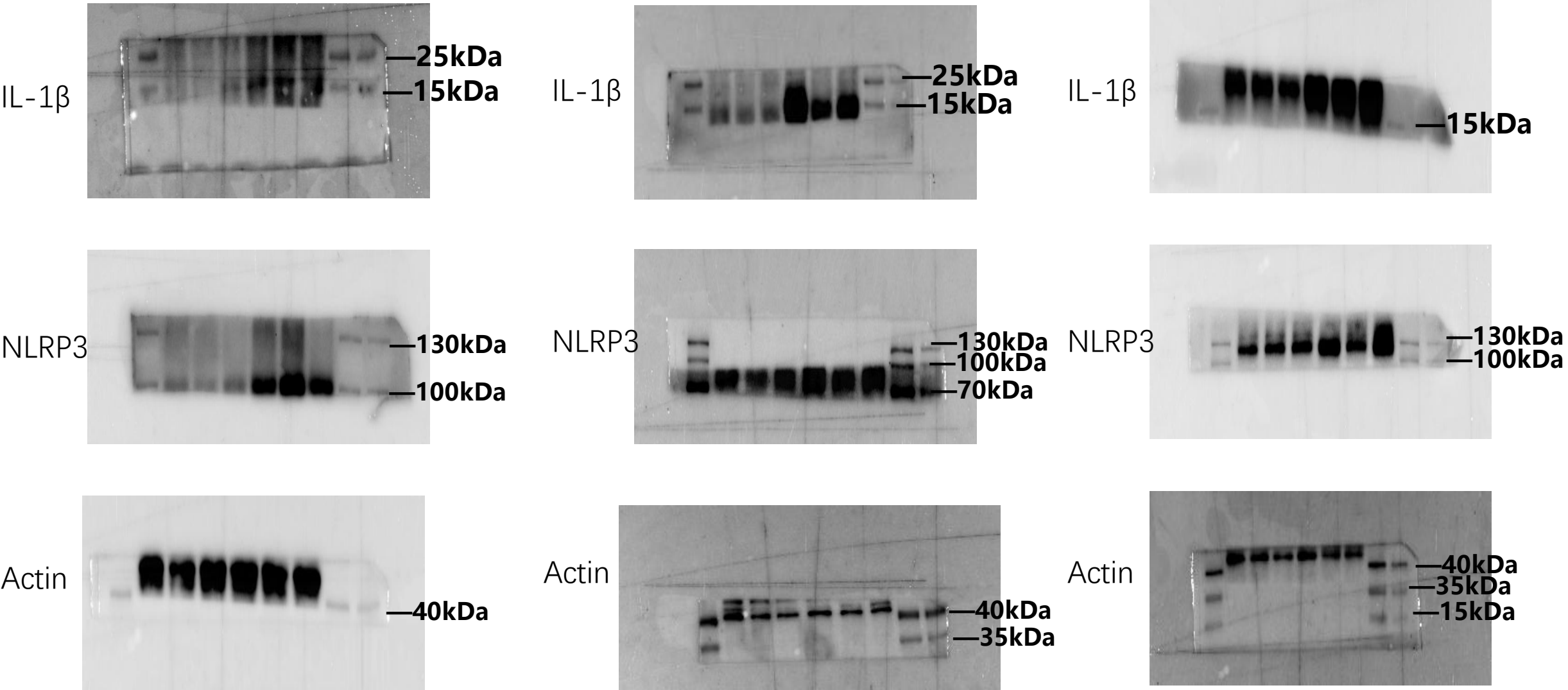

Fig3.A --NLRP3、 CD80

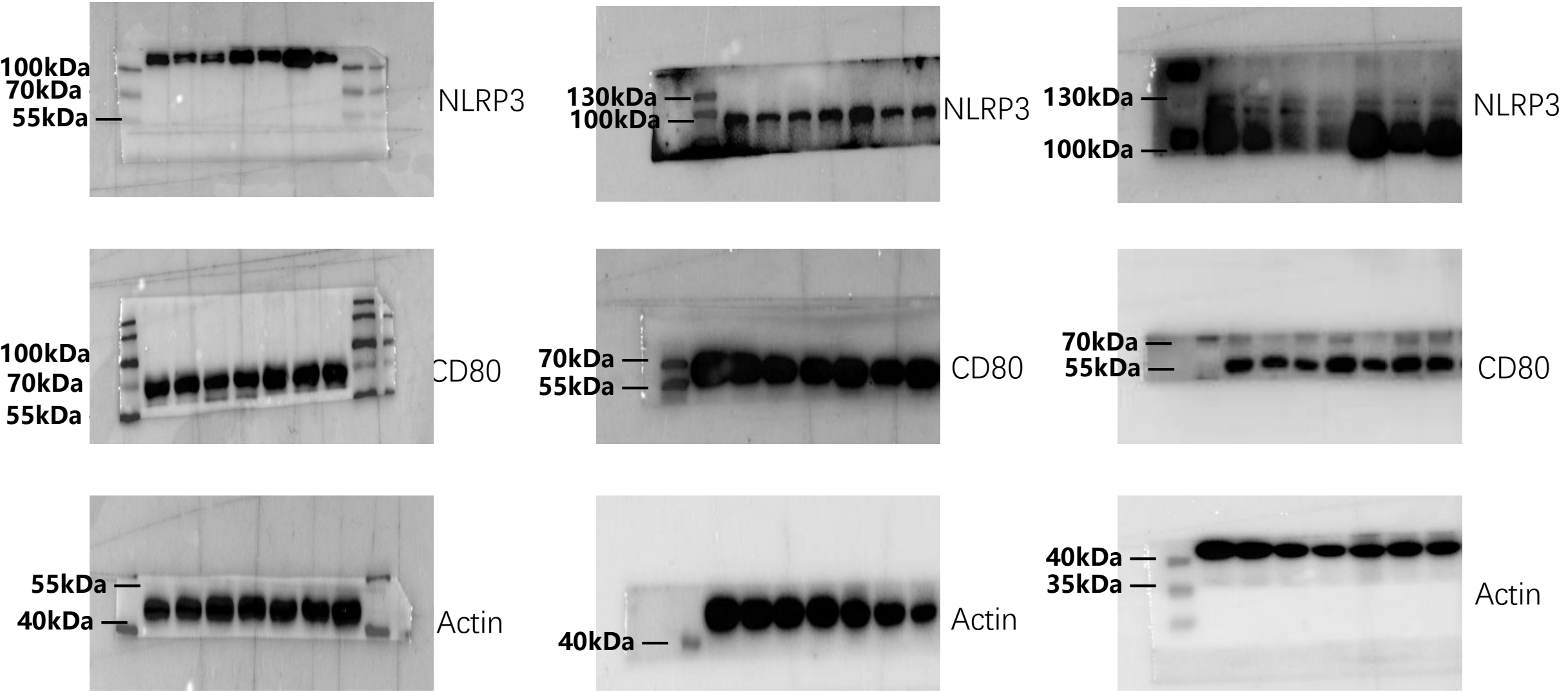

Fig4.G--PDH

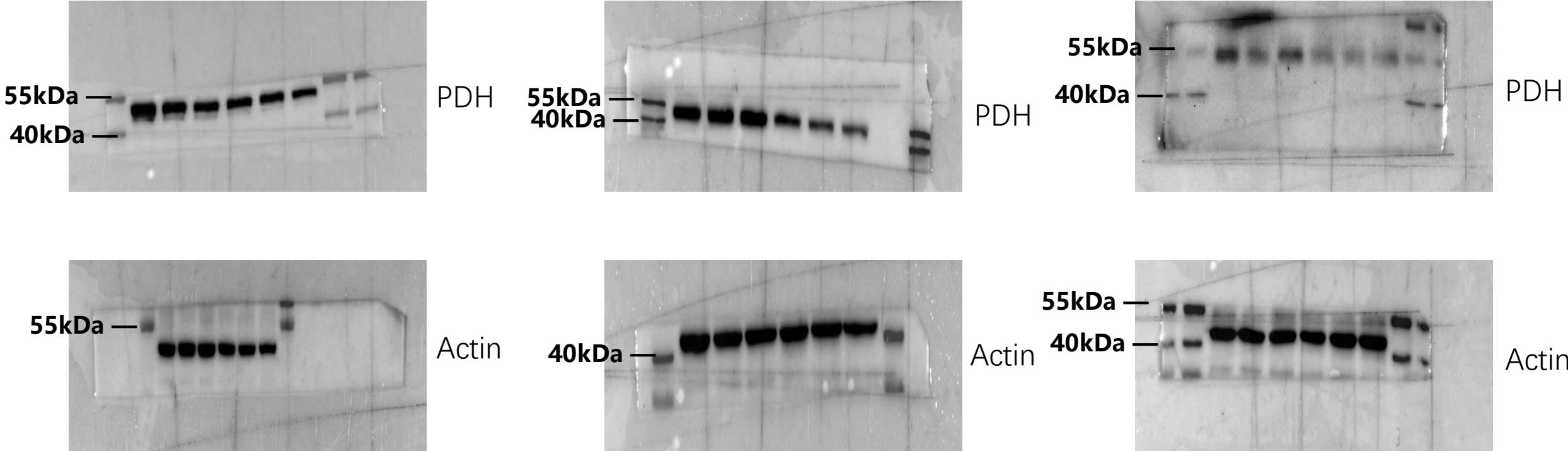

Supplement: S1 File — (PDF) [file pone.0328794.s001.pdf]

Fig.2B HE

WT

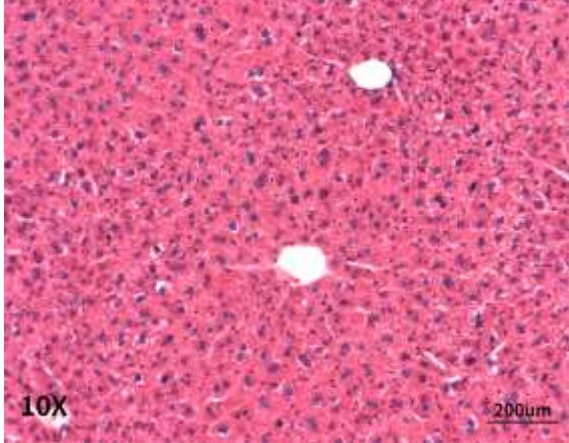

ob/ob

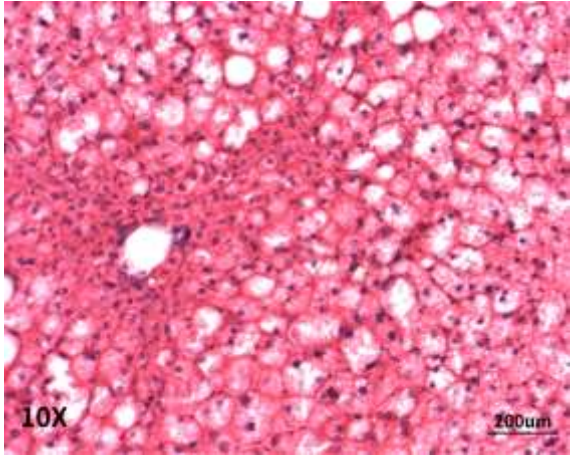

WT

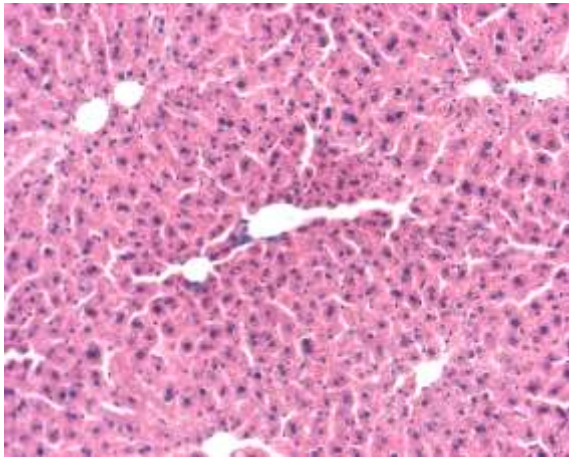

ob/ob

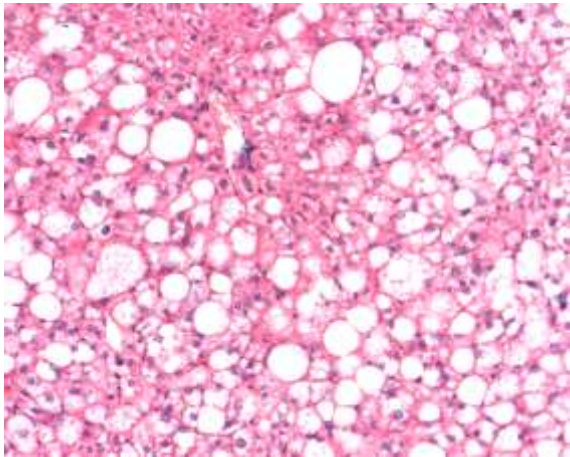

WT

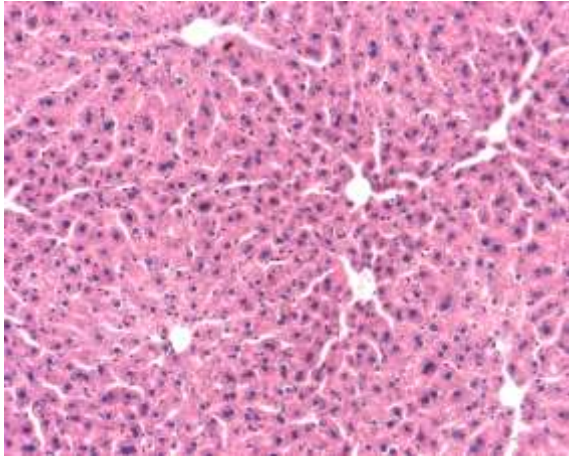

ob/ob

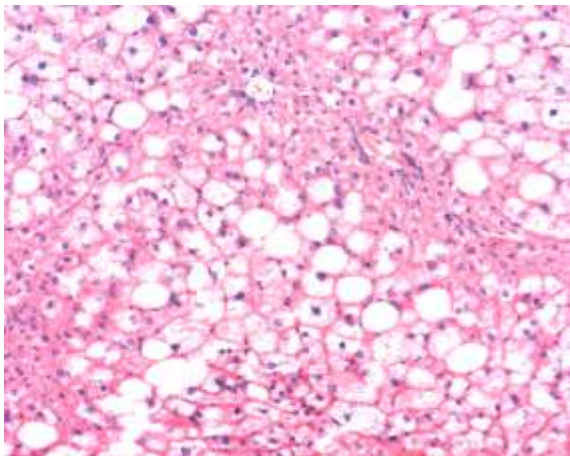

Fig.2B Oil red  
staining

WT

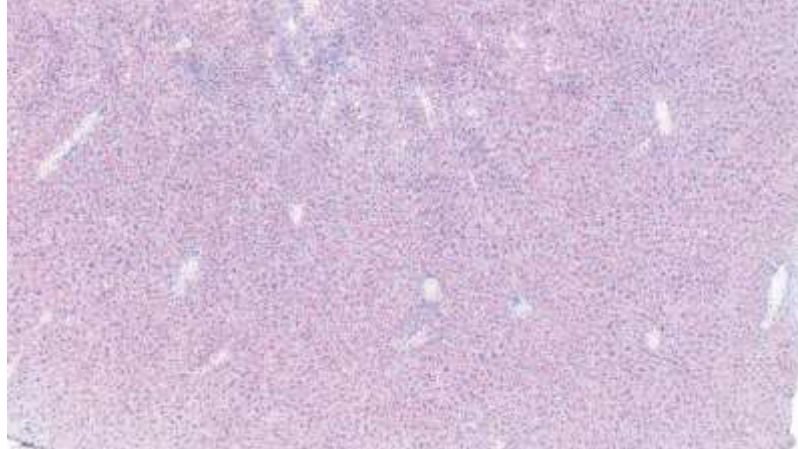

ob/ob

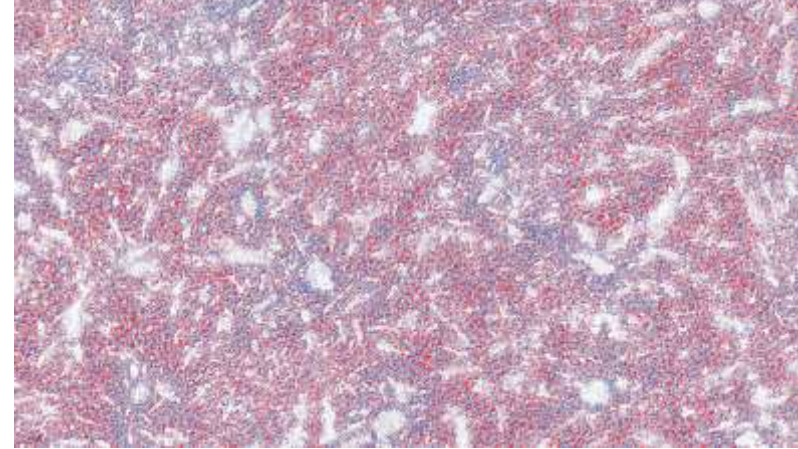

WT

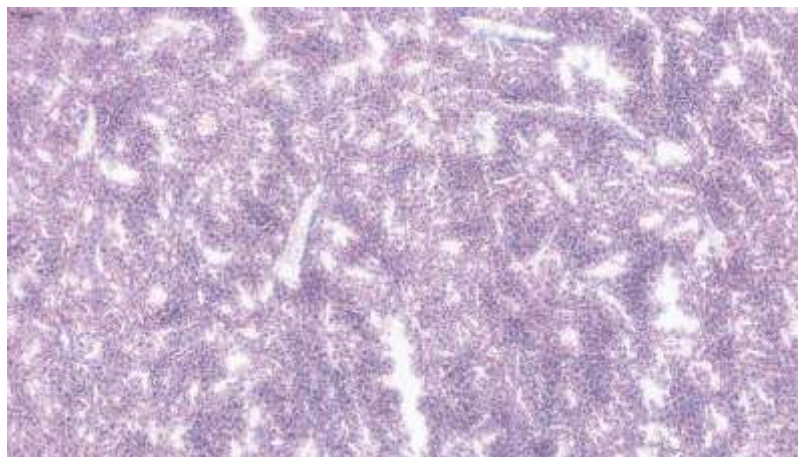

ob/ob

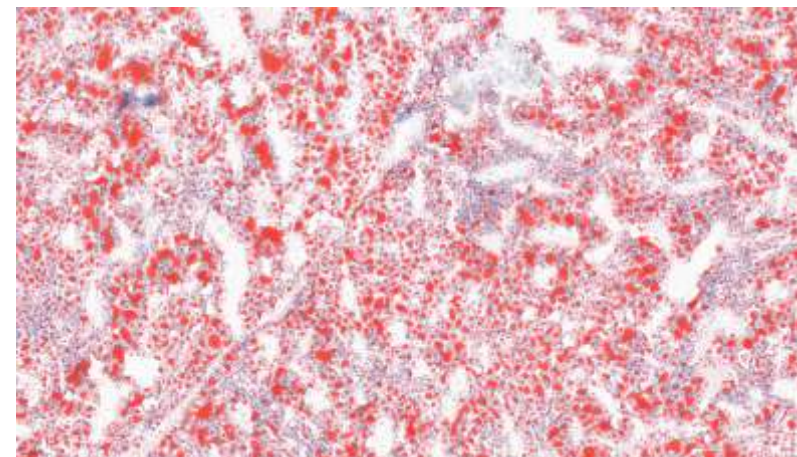

WT

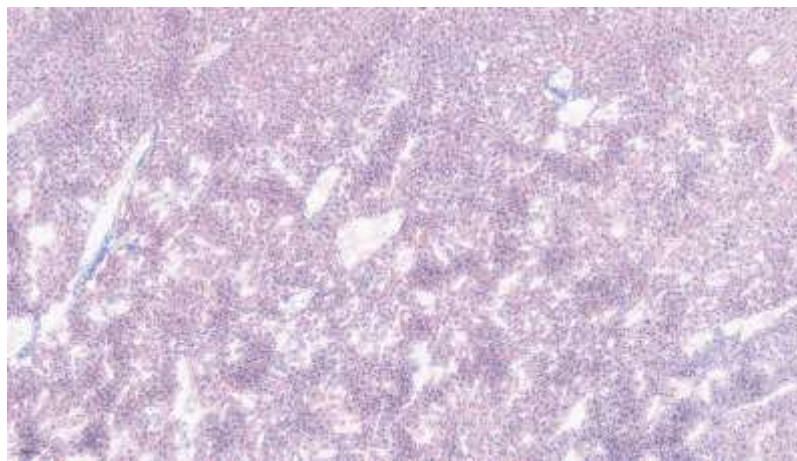

ob/ob

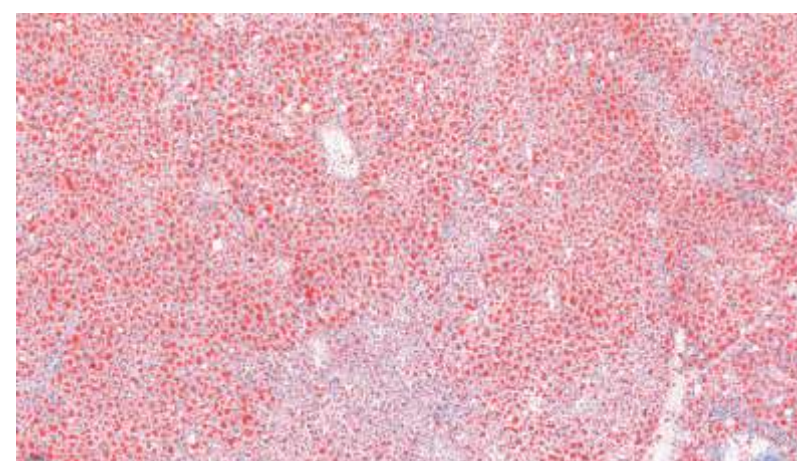

Supplement: S2 File — (PDF) [file pone.0328794.s002.pdf]
